# PReS-FINAL-2085: The p38-mediated rapid downregulation of cell surface gp130 expression impairs IL-6 signaling in the synovial fluid of juvenile idiopathic arthritis patients

**DOI:** 10.1186/1546-0096-11-S2-P97

**Published:** 2013-12-05

**Authors:** N Honke, K Ohl, A Wiener, N Wagner, S Wüller, K Tenbrock

**Affiliations:** 1 Pediatrics, RWTH Aachen University, Universitätsklinikum, Aachen, Germany

## Introduction

In patients with juvenile idiopathic arthritis (JIA) high levels of IL-6 are present in the serum and synovial fluid (SF). IL-6 signaling plays an important pro-inflammatory role but is restricted by regulatory mechanisms such as reducing the cell surface availability of the signal-transducing chain of the IL-6 receptor (gp130).

## Objectives

The aim of this study was to determine whether the inflammatory environment in the arthritic joint has an impact on monocytic gp130 surface expression and the extent to which regulatory processes in the SF can be transferred to an *in vitro *model.

## Methods

Flow cytometry and live-cell imaging were used to measure the cell surface expression and internalization of gp130. STAT3 phosphorylation was monitored by flow cytometry and western blotting.

## Results

The level of cell surface gp130 expression on SF monocytes was reduced compared to peripheral blood (PB) monocytes from patients with JIA. This reduction could be reproduced by stimulating PB monocytes from healthy donors with SF and was dependent on p38 MAPK. The induction of p38 by IL-1β in PB monocytes interfered with IL-6 signaling due to the reduced cell surface expression of gp130.

## Conclusion

The results suggest that p38-mediated pro-inflammatory stimuli induce the downregulation of gp130 on monocytes and thus restrict gp130-mediated signal transduction. This regulatory mechanism could be relevant in the inflamed joints of patients with JIA.

## Disclosure of interest

None declared.

